# Family correlates of behavioral problems among adolescents in Rwanda

**DOI:** 10.1371/journal.pone.0314507

**Published:** 2025-02-10

**Authors:** Eugene Harerimana, Jean d’Amour Muziki, Eric Nshimyumuremyi, Thaoussi Uwera, Augustin Nshimiyimana, Jean Mutabaruka

**Affiliations:** 1 Department of Clinical Psychology, College of Medicine and Health Sciences, University of Rwanda, Kigali, Rwanda; 2 Department of Health Informatics, College of Medicine and Health Sciences, University of Rwanda, Kigali, Rwanda; Fondazione Policlinico Universitario Agostino Gemelli IRCCS, Universita’ Cattolica del Sacro Cuore, ITALY

## Abstract

**Background:**

Globally, 20% of adolescents exhibit behavioral problems. Behavioral problems are associated with individual and environmental factors. However, little is known about the contribution of the nuclear family to the development of behavioral problems in adolescents from sub-Saharan Africa. This study aimed to explore family-based correlates influencing behavioral problems among adolescents in Rwanda.

**Methods:**

With an institutional-based cross-sectional study design, a convenience sample of 158 participants {107 males and 51 females; Mean age (M) = 16.96, Standard Deviation (SD) = 1.86; age ranging from 13 to 23 years} was selected in secondary schools in the Nyarugenge district. Participants filled out Behavioral Problems Scale (BPS), Child and Adolescent Trauma Screen (CATS), University of California, Los Angeles Loneliness Scale (UCLA Loneliness Scale), Multidimensionality of Perceived Social Support Scale (MSPSS), Multidimensional Neglectful Behavior Scale (MNBS), and Paediatric Quality of Life Enjoyment and Satisfaction Questionnaire (PQ-LES-Q) to record pertinent scores. Socio-demographic information was also collected. SPSS version 24 was used for statistical analysis.

**Results:**

Females exhibited more behavioral problems than males. Child and adolescent trauma (β = 0.705, t = 8.21, p < .001) and neglect (β = 0.147 t = 2.15, p < .05) were two significant family correlates in our sample. Poor quality of life enjoyment and satisfaction, loneliness, and poor parental perceived social support were not identified as family-based factors that influence behavioral problems in our sample.

**Conclusions:**

Results highlighted the importance of implementing family and community-based interventions to sustain family well-being, change parenting behaviors, and help children and adolescents adopt positive behaviors.

## Background

According to the definition by the World Health Organization, adolescence is generally a period approximately between the ages of 10 and 19 [1]. However, to improve popular understanding of this period of adolescent growth and facilitate long-term investments in a wider range of contexts, adolescence was recently defined as a period between 10 and 24 years [2]. It is therefore divided into early (10–14 years of age), middle (15–16 years of age), and late adolescence (17–21 years of age and beyond) [3]. This period of development between childhood and adulthood is marked by a high rate of biological and emotional change and maturity [4]. It is widely acknowledged that adolescence is a crucial life stage during which numerous behavioral habits that influence one’s current health status and future health outcomes are formed [5].

Adolescent behavioral issues are a worldwide concern [6]. They refer to individuals’ actions that influence their daily living conditions or surroundings by probably creating a health risk for other people or themselves [7]. Adolescents with behavioral issues typically aggress against other people and animals, damage properties, purposefully break rules, react with anger and irritability, and consistently disobey their parents and authorities [8–12]. Externalizing behaviors (e.g., physical, verbal, and relational aggression, vandalism, theft, and disobedience) [13] and internalizing behaviors, also known as lashing out at oneself, (e.g., depression, eating disorder, anxiety, and substance use) [14] are the two types of behavioral problems in adolescents.

Behavioral problems are common in adolescents [14,15]. In 2001, the global estimate of behavioral problems in adolescents accounted for 20% [16]. Equivalently, in some countries like Australia and Brazil, the prevalence of behavioral problems in adolescents is respectively 13.4%[17] and 20.4% [18], while it is respectively 2% to 9.61% and 17.6% in school children in Uganda[19] and China [20]. Adolescents with behavioral problems are more likely to experience negative outcomes later in life, such as school dropout, social and health impairments [21], becoming a parent at an earlier age [22–24], suicidal attempts [22–24], psychiatric disorders [22,24,25], unemployment [23,24], substance abuse [22–24], separation or divorce [26], and criminality [27].

At the broadest level, many authors have noted that behavioral problems in adolescents are influenced by inconsistent discipline, gender, and parental deviance or mental disorder [28], adverse childhood experiences (verbal and sexual abuse) and misusing routinely substances [29], lower self-control and social skills, a higher frequency of physical abuse and maternal neglect [30], a mother’s attitude (poor maternal response to the child’s needs due to postnatal psychological stress) [31], family patterns[32], and the influence of other adolescent risk behaviors, neighborhood, peers, and school [33]. Similarly, the comprehensive theoretical model of problem behaviors and a developmental theory of reckless behavior suggested that behavioral problems in adolescents are influenced, respectively, by family socioeconomic status [34] and sensation-seeking and adolescent egocentrism [35], while problem behavioral theory refers to behavioral problems in adolescents as any learned behavior that is functional, purposeful, and instrumental in achieving goals [36].

Furthermore, studies emphasized the role of family members in protecting the psychological well-being of adolescents and children[32,37]. However, we should not ignore the role of the family in magnifying behavioral problems in adolescents [38]. Therefore, authors have investigated the contribution of some family correlates such as household food insecurity [39], the quality of family members’ support[32], positive parent–child relationships, religiosity, school quality, and social support networks [40], in the development of behavioral problems among adolescents. However, research on similar family-based factors such as childhood trauma [41], neglect [42], social isolation and loneliness [43], perceived social support [44], and the quality of life enjoyment [45], in the sample of adolescents from sub-Saharan African countries like Rwanda is lacking. According to different authors’ and Maslow’s Humanistic Theory, these factors fall into deficiency needs such as security, which contributes to preventing neglect and childhood trauma [46–48]; friendships and social networks, which contribute to avoiding isolation and loneliness [47,49]; a personal relationship with family, which contributes to increased parental perceived social support [47,50]; and fulfillment of physiological and psychological needs, which leads to a high level of quality-of-life enjoyment and satisfaction [47]. In spite of that, it is well known that satisfying the needs of love, security, and personal relationships with the family, as well as physiological and psychological needs, can prevent unpleasant feelings and behavioral problems [47,50].

Childhood trauma is mainly understood as a violent family environment where a child or adolescent can be a witness or victim of the violence [51], while neglect occurs when a parent or other caregiver persistently fails to meet a child’s physical and/or psychological needs, which is likely to have a major negative impact on the child’s development or health [52]. Apart from individual traits, geographic location, and peer experiences [53,54], parent and family contextual factors (e.g., parental health, parental divorce, family lifestyle, quality of parent-child relationship, family socioeconomics, etc.) are root causes for loneliness among adolescents[55,56]. Paediatric quality of life enjoyment and satisfaction are increased when adolescents have enough time within the family environment to talk to parents about their problems [57], while family-based perceived social support (support from the parents) protects children and adolescents from engagement in health-harming behaviors and increases the likelihood of academic success and interpersonal relationship [58].

Overall, studies on the mental health of children and adolescents in Rwanda are still too scarce to inform the research community, decision-makers, and policymakers. Few studies have focused on the perceptions of mental health problems in HIV/AIDS-affected children and adolescents [59], perceptions of parents and adolescents on the effectiveness of communication training programs [60], the risk and protective factors for alcohol and other drug abuse among adolescents and their parents [61], abuse facing adolescents heads of households [62],post-traumatic stress disorder (PTSD) in mothers and mental health outcomes among their children [63], home visiting interventions to promote mental health in children affected by HIV [64], and the impact of a foster family on the mental health of more vulnerable children [65]. A recent study in a similar context provided a general image of adolescent mental health needs in Rwanda, but there is still a lack of scientific evidence on the role of family dysfunction in psychological problems among adolescents [66].

**Fig 1:**
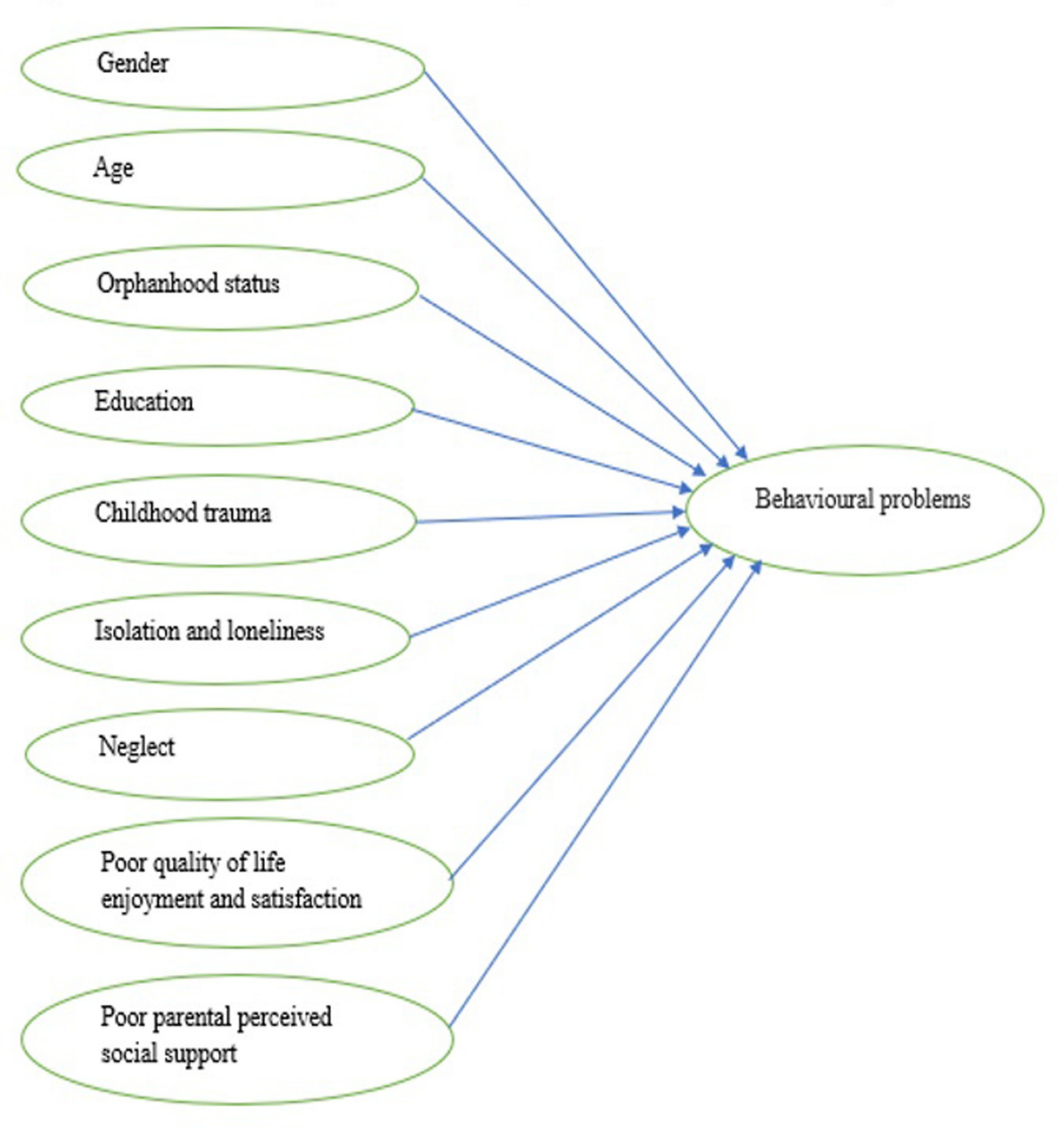
A model diagram of family correlates of behavioral problems in adolescents.

This study intends to explore family correlates of behavioral problems among adolescents who were identified across different secondary schools in Nyarugenge District (Kigali-Rwanda) to attend counseling sessions. Socio-demographic variables (Fig 1) were age, sex, level of education, and orphanhood status. First, we expect a significant difference in behavioral problems across sociodemographic variables. Second, we anticipate that a higher rate of childhood trauma, isolation and loneliness, neglect, poor quality of life enjoyment and satisfaction, and poor parental perceived social support will be associated with a higher rate of behavioral problems (Fig 1).

## Methods

### Study design, sample, and procedures

The study was designed as an institutional-based cross-sectional study. The convenience sample, composed of 158 adolescents (107 males and 51 females; M = 16.96, SD = 1.86), with ages ranging from 13 to 23, was selected in all secondary schools located in Nyarugenge District from January up to March 2022. The total target population was composed of 209 students (138 males and 71 females) who were identified by school counselors as students with one or more disruptive behaviors and therefore would attend counseling sessions. The common disruptive behaviors were struggle with educators and relationships, aggression, theft, loss of school interest, inappropriate sexual behaviors, and self-isolation. However, those eligible were 158 students who were registered in both lower and upper secondary schools. As inclusion criteria, we recruited subjects to attend counseling sessions who were aged between 13 and 23 years, willing to participate in the study, living with one or both parents or a guardian, and able to read and respond to the self-report questions on the research instruments. Participants with parents in detention centers, communication problems, severe mental disturbances, or another chronic illness that would affect their judgment were excluded from the study. So, excluding adolescents with parental incarceration helped understand well the contribution of the family instead of understanding the contribution of parental incarceration to the poor mental health of the participants, denoted here as behavioral problems. Regarding sampling calculations, no a priori power calculations were carried out because of the convenience sampling approach.

Approval for conducting the study (No. 389/CMHS IRB/2021) was obtained from the Institutional Review Board of the University of Rwanda, CMHS (IRB-CMHS). Before starting data collection, we explained the study’s research objectives and ethical guidelines to the participants. Those aged 18 years and older provided written consent to participate in the study, while we obtained consent from the parents or guardians of the minors. We also assured participants that it was their right to withdraw from the study if they did not want to participate or if they changed their mind. Research questionnaires were granted for non-commercial applications and were then translated into Kinyarwanda by three Kinyarwanda, French, and English-speaking psychologists, while the reverse translation was done by two psychologists who have proficiency in Kinyarwanda, French, and English [67]. The participants’ psychological distress in response to the research questionnaires was addressed in a clinical setting with the close collaboration of trained school counselors.

### Measurements

The Behavioral Problems Scale (BPS) assessed behavioral problems in adolescents on a 5-point Likert rating scale from 1 (never) to 5 (most often), where a higher score indicates that the participant had severe behavioral problems [7]. Cronbach’s alpha was .64 in the current study. Fortunately, it is between 0.6 and 0.7, indicating an acceptable level of reliability for our questionnaire [68].

The Child and Adolescent Trauma Screen (CATS) was used to measure childhood trauma [69]. This questionnaire consists of twenty items, which are scored on a 4-point Likert scale ranging from 0 (never) to 3 (almost always). A cut-off score greater or equal to 21 indicates that an individual has a clinically relevant level of symptoms of childhood trauma [69]. When using CATS in our sample, this study reported a Cronbach’s alpha of .91.

Feelings of loneliness and social isolation were measured by the University of California, Los Angeles Loneliness Scale (UCLA Loneliness Scale) [70]. This questionnaire comprises 20 items designed to measure one’s subjective feelings of loneliness as well as feelings of social isolation, where a participant is required to rate each item on a 4-point Likert scale from 1 (never) to 4 (often) [70]. The current study demonstrated a Cronbach’s alpha of .82.

Perceived social support was assessed by the Multidimensionality of Perceived Social Support Scale (MSPSS), which is composed of 12 items [71].This questionnaire captures the multidimensionality of perceived social support through the measurement of items for social support from friends, family, and one’s significant other [71]. Each item is rated on a 7-point Likert scale ranging from “very strongly disagree” to “very strongly agree.” A higher score indicates a higher level of perceived social support [71]. In the current sample, Cronbach’s alpha was .90.

The Multidimensional Neglectful Behavior Scale (MNBS) was used to assess the level of experienced neglect [72]. This is an 8-item questionnaire rated on a 4-point Likert scale ranging from strongly disagree (1) to strongly agree (4). Cronbach’s alpha coefficient for our sample was .72.

Life satisfaction was measured by the Paediatric Quality of Life Enjoyment and Satisfaction Questionnaire (PQ-LES-Q) [73–75]. It is a 15-item, self-administered questionnaire for children and adolescents, where each item is rated on a 5-point Likert scale from 1 (very poor) to 5 (very good) [73–75]. In the current sample, the Cronbach’s alpha value was .80.

### Statistical analysis

SPSS version 24 was used to compute descriptive statistics, comparative analyses, internal consistency of questionnaires, correlations, regression with a p-value of α = 0.05 retained, and an independent t-test and its extension (one-way ANOVA). An independent t-test was used to examine whether there was a significant difference in behavioral problems across personal characteristics such as age, gender, and level of education, while a one-way ANOVA was used for comparison of participants’ behavioral problems in the different groups of orphanhood status. Before computing multiple regression analyses to measure the contribution of each independent variable to the dependent variable, the major assumptions of this analysis were adequately considered. Therefore, a normal distribution was verified, the dependent variable had homoscedasticity, multicollinearity was not a problem for the predictor variables, the tolerance values were greater than 0.4, and there were no outliers. Cook’s distance value did not exceed 1 (minimum value = 0, maximum value = 0.096).

## Results

### Analysis of socio-demographic characteristics

The sample was composed of 158 participants (M = 16.96, SD = 1.86), where males (67.7%) outnumbered females (32.3%). Considering growth stages, most of the participants (67.7%) were in early and middle adolescence (10–17 years), while those in late adolescence (18 years and older) represented 32.3% of the total sample. Regarding orphanhood status, many participants were non-orphans (68.3%), followed by paternal orphans (24.7%). Then, double orphans were 3.8%, while maternal orphans were 3.2%. Finally, 84.2% of the participants were registered in lower secondary school during the study period, while 15.8% were registered in upper secondary school. The results of the sociodemographic analysis are presented in Table 1.

**Table 1:** Characteristics of participants.

Gender	n	%
Males	107	67.7
Females	51	32.3
Total	158	100.0
**Categories of participants by age**	**n**	**%**
Early and middle adolescence (10–17 years)	107	67.7
Late adolescence (18 years and above)	51	32.3
Total	158	100.0
**Orphanhood status**	**n**	**%**
Non-orphan	108	68.4
Paternal orphan	39	24.7
Maternal orphan	5	3.2
Double orphan	6	3.8
Total	158	100.0
**Education**	**n**	**%**
Lower secondary school	133	84.2
Upper secondary school	25	15.8
**Total**	**158**	**100.0**

### Normality testing and descriptive analysis of study variables

Table 2 was primarily performed to determine the kurtosis and skewness of our scale’s distribution. Results from Table 2 showed that the study scales are found within the normal range, which assumes that skewness and kurtosis values should fall between *-*2 and +2 [76]. As our data were normally distributed, it was advisable to use parametric statistical tests for further analysis [77]. Further, in Table 2, the mean age of the participants was 16.96 (SD = 1.861) while the mean scores for BP, CAT, PQLES, loneliness, neglect, and PSS were respectively 21.3 (SD = 5.16), 18.07 (SD = 11.18), 19.30 (SD = 11.3), 42.89 (SD = 9.31), 15.19 (SD = 4.259), and 61.56 (SD = 15.11).

**Table 2:** Descriptive analysis of study variables.

	Age	BP	CATS	PQLES	Loneliness	Neglect	PSS
**Mean**	16.96	21.30	18.07	52.51	42.89	15.19	61.56
**Std. Deviation**	1.861	5.168	11.182	11.308	9.316	4.259	15.115
**Skewness**	0.650	0.656	0.863	-0.473	0.152	0.182	-0.995
**Kurtosis**	0.531	1.358	0.591	0.403	-0.092	-0.656	0.780

### Comparison of behavioral problems across different personal characteristics

According to Table 3, an independent t-test revealed no statistically significant differences in behavioral problems between lower secondary school and upper secondary school (t (156) = .025, p = .98), or between early, middle, and late adolescence (t (156) = -.41, p = .68). There were, however, statistically significant differences in behavioral problems between males and females (t (156) = -3.06, p = .003), with females having more behavioral problems (M = 23.07; SD = 5.31) than males (M = 20.46; SD = 4.90). In addition, when we performed a one-way ANOVA test, no significant differences were detected in behavioral problems among non-orphans, paternal orphans, maternal orphans, and double orphans (F (3,154) = .128, p = .943). Results from a one-way ANOVA are presented in Table 4.

**Table 3:** Results from independent samples test.

				Levene’s Test for Equality of Variances		t-test for Equality of Means		
Groups of participants	N	Mean	SD	F	Sig.	t	df	Sig. (2-tailed)
Males	107	20.46	4.90	.019	.89	-3.06	156	.003
Females	51	23.07	5.31					
Early and middle adolescence	107	21.19	5.16	.013	.90	-.41	156	.68
Late adolescence	51	21.55	5.22					
Lower secondary school	133	21.30	5.39	1.87	.17	.025	156	.98
Upper secondary school	25	21.28	3.88					

**Table 4:** One-way ANOVA results.

				Test of Homogeneity of Variances		ANNOVA	
Orphanhood	N	Mean	SD	Levene’s Statistic	Sig.	F	Sig.
Non-orphan	108	21.30	5.20	.54	.65	.128	.943
Paternal orphan	39	21.18	5.01				
Maternal orphan	5	20.8	5.50				
Double orphan	6	22.5	6.47				

### Bivariate corralation analyses

In Table 5, bivariate correlation analyses showed that behavioral problems (BP) is positively correlated with child and adolescent trauma (CAT) (r = .68, p < .001), loneliness (r = .46, p < .001), and neglect (r = .35, p < .001). Besides, BP is negatively correlated with quality of life Enjoyment and satisfaction (PQLES) (r = -.45, p < .001) and perceived social support (PSS) (r = -.33, p < .001). These findings confirm a plausible association between the dependent and independent variables.

**Table 5:** Correlation between BP, CAT, PQLES, loneliness, neglect, and PSS (N = 158).

	1	2	3	4	5	6
**BP**	1	.688^**^	-.450^**^	.464^**^	.353^**^	-.339^**^
**CAT**		1	-.710^**^	.588^**^	.336^**^	-.469^**^
**PQLES**			1	-.573^**^	-.397^**^	.573^**^
**Loneliness**				1	.487^**^	-.591^**^
**Neglect**					1	-.478^**^
**PSS**						1

**. Correlation is significant at the 0.01 level (2-tailed).

*. Correlation is significant at the 0.05 level (2-tailed).

1: BP, 2: CAT, 3: PQLES, 4: Loneliness, 5: Neglect, 6: PSS.

### Significant family correlates of behavioral problems among the participants

The outcome variable (behavioral problems), as shown in Table 5, was highly correlated with independent variables such as child and adolescent trauma, padiatric quality of life enjoyment and satisfaction, loneliness, neglect, and perceived social support. These independent variables were subsequently entered into a multiple regression model to test those with a significant association with BP. As presented in Table 6, only two independent variables, namely CAT (β = 0.705, t = 8.21, p < .001) and Neglect (β = 0.147, t = 2.15, p < .05), predicted positively BP. This finding means that a higher level of neglect and trauma among adolescents increases the level of behavioral problems in adolescents.

**Table 6:** Results from multiple regression analysis.

	Unstandardized Coefficients		Standardized Coefficients	t	Sig.
	B	Std. Error	Beta		
(Constant)	7.250	3.887		1.865	0.064
CAT	0.326	0.040	0.705	8.215	0.000
PQLES	0.061	0.041	0.133	1.494	0.137
Loneliness	0.039	0.046	0.071	0.860	0.391
Neglect	0.179	0.083	0.147	2.156	0.033
PSS	0.009	0.027	0.027	0.349	0.727

a. Dependent Variable: BP.

## Discussion

The purpose of the study was to explore family correlates of behavioral problems among adolescents identified across different secondary schools in Nyarugenge District (Kigali-Rwanda) who attend counseling sessions. First, our study found that girls exhibit more behavioral problems than boys. Despite the opposite results from the study conducted on preadolescents aged 11–12 years in Pakistan [78], the study conducted on Indian adolescents revealed that females are more affected by behavioral problems than males [31]. The existence of behavioral problems in our female participants may be explained by family processes [79], which can include family dysfunction [80], maltreatment [81], and increased levels of conflict, deviance, and discord [82]. In addition, girls are generally more strongly affected than boys by an unpleasant family atmosphere or another adverse family environment [83].

Then, the current study sought to investigate the significant family correlates of behavioral problems in the sample. Child and adolescent trauma predicted positive behavioral problems in our participants in this regard. This finding means that experiencing more childhood trauma increases behavioral problems in our participants. This is consistent with the study conducted on American college students from a Midwestern university, which showed that childhood trauma predicted behavioral problems among adolescents, where experiential avoidance and mindfulness processes played a role in mediation [84]. After that, child or adolescent neglect was proven to be a positive determinant of behavioral problems. This finding means that children or adolescents who were raised by neglectful parents in their respective families experience more behavioral problems or are at higher risk of developing behavior problems. Other researchers have found that neglect predicts behavioral problems in children and adolescents [42,85–87]. In contrast to our students, unwholesome familial dynamics such as rejection, physical and verbal conflict, negative communication, harsh discipline, and disengagement are other factors that increase behavioral problems in adolescents [88].

Finally, some family-based factors associated with behavioral outcomes are not significant in our sample. Those are poor quality of life enjoyment and satisfaction [89], loneliness [90,91], and poor parental perceived social support [92], highlighted by previous studies as significant predictors of poor mental health outcomes, including behavioral problems for adolescents. Ongoing interventions like the psychosocial support provided in youth-friendly facilities, the establishment of an equipped school-based mental health program, empowerment of communities as a whole to address the root causes and risk factors for mental health in adolescents, special assistance provided to pregnant adolescents and teen mothers, and importation and adaptation of non-local successful digital mental health interventions with similar settings as Rwanda [66] might have prevented behavioral outcomes associated with loneliness, poor parental perceived social support, and poor quality of life enjoyment and satisfaction in our sample. Another reason why poor quality of life enjoyment and satisfaction, isolation and loneliness, and poor parental perceived social support were not significant in this study can be explained by Maslow’s humanistic theory. This theory argues that getting the minimum required physiological and psychological needs, having meaningful friendships and social networks, and experiencing positive family relationships can, respectively, lead to a standard level of quality-of-life enjoyment and satisfaction, prevent loneliness and isolation, and improve parental perceived social support [47]. However, mental health for adolescents is not portrayed as a priority issue for immediate attention, even though Rwandan children and adolescents are still facing various challenges such as adverse childhood experiences induced by the 1994 Genocide against the Tutsi, family conflicts, online abuse, poverty, risky behaviors, substance abuse and addiction, and unintended teenage pregnancies associated with violence against children and women [66].

### Implication for practice and limitations

This study provides information about the psychological well-being of schoolgirls or schoolboys who are frequently considered unmanageable due to a stressful family background that is beyond their control. The participants have been struggling with the kinds of behavioral problems that emerge from family dysfunction, such as trauma and neglect occurring during childhood or adolescence. Recent studies showed that family and community-based interventions like positive parenting programs incorporating psychosocial interventions can change parenting behaviors vis-à-vis their parenting styles, which can in turn help children and adolescents in the treatment and prevention of behavioral problems and the adoption of positive behaviors [93–95]. Therefore, our study findings benefit programs targeting family cohesion, psychological well-being, financial stability, healthy social interaction, safety, and the good health of all family members. In fact, theories on family well-being explain the contribution of these measures to building family well-being [96]. As a result, the level of resilience among adolescents and children will be strengthened, and their behavioral problems will be prevented and treated within their respective families by focusing on females because they were highlighted by the study as a more vulnerable group.

Despite the importance of the study findings for the well-being of the family in general and for adolescents in particular, there were limitations to be considered for future studies. The first limitation was a concern about the generalizability of the findings. The current study adopted an institutional-based cross-sectional study design with a convenience sample composed of urban students instead of rural students. Relying on an unrepresentative sample that was chosen through non-random sampling can weaken the ability to generalize the findings. Therefore, a larger study with probabilistic sampling techniques may improve conclusions and inferences for appropriate psychosocial support. Second, our study was conducted on a sample of participants from urban areas. This means that the findings do not reflect the reality of adolescents in rural areas. Consequently, a similar study with participants from both areas is required to test differences or similarities. Third, our study focused on adolescents who were enrolled in their respective schools. Adolescents who dropped out were not included in this study. So, considering them for future studies can improve the findings. Finally, participants with parents in detention centers were not included in this study. So, the particularity of participants with incarcerated parents should be investigated to enrich the scientific evidence.

## Conclusion

As revealed by the study findings, schoolgirls exhibit more behavioral problems than schoolboys. This problem was associated with childhood trauma and neglect. Results highlighted the importance of implementing family and community-based interventions to sustain family well-being, change parenting behaviors, and help children and adolescents adopt positive behaviors.

## Supporting information

S1 Dataset https://doi.org/10.6084/m9.figshare.22298245.v1.
(SAV)
